# Toll-Like Receptor 4 (TLR4) Expression Affects Schwann Cell Behavior *in vitro*

**DOI:** 10.1038/s41598-018-28516-5

**Published:** 2018-07-25

**Authors:** Huanhuan Zhang, Zhiwei Shao, Yun Zhu, Lingyu Shi, Zhihao Li, Rui Hou, Chunwang Zhang, Dengbing Yao

**Affiliations:** School of Life Sciences, Key Laboratory of Neuroregeneration, Co-innovation Center of Neuroregeneration, Nantong University, Nantong, Jiangsu 226019 P.R. China

## Abstract

Peripheral nerve injury can result in the decreased quality of life and bring us economic burden on society and individuals. Wallerian degeneration (WD) is critical for nerve degeneration and regeneration, but the mechanisms of WD are still elusive. Here, we report the effect of Toll-like receptor 4 (TLR4) on cultured Schwann cells (SCs) *in vitro*. The data showed that TLR4 expression was up-regulated after sciatic nerve injury of rat. TLR4 was expressed in cultured SCs. Enhanced or silenced expression of TLR4 affected SC proliferation, migration, apoptosis and relative gene expression. Furthermore, altered expression of TLR4 resulted in expression changes in c-Jun, ERK and catenin but not AKT and c-Fos pathways in SCs. These results suggested that TLR4 may be an important effective target in peripheral nerve degeneration and/or regeneration during WD in future investigations.

## Introduction

In the peripheral nervous system (PNS), Wallerian degeneration (WD) occurs rapidly in the PNS, following axonal degeneration and regeneration^1–4^. Schwann cells (SCs) may play important roles in nerve injury, repair and regeneration in response to various stimuli of WD. Peripheral nerve injury has been studied extensively. A great number of genes are expressed differentially during this process, but the molecular mechanisms are not completely clear. Therefore, studying the factors which regulate peripheral nerve injury, repair and regeneration may explain the mechanisms of WD^5,6^.

In our previous study, we reported on the presence of gene expression pathways and signal flow regulated by key factors, such as claudins, transforming growth factor, beta 1 (TGF-β1), secreted phosphoprotein 1 (SPP1), Toll-like receptors (TLRs), and Fas Ligand Gene (Faslg), during WD in nerve stumps after sciatic nerve injury in the rat using microarray analysis^7–11^. In this study, we reported the role of TLR4 on cell migration and proliferation, cell apoptosis, and signal pathways in Schwann cells *in vitro*. Toll-like receptors (TLRs) are responsible for the detection of damage-associated molecular and pathogen-associated molecular patterns^12–14^. TLR4 is the most extensively studied TLR and recognizes lipopolysaccharides. TLR4 is expressed in innate immune cells. TLR4 is also expressed in neurons and central nervous system cells, including astrocytes and microglia. It is involved in nervous system development and regulates the differentiation and proliferation of adult neuronal precursor cells. Activation of TLR4 is facilitated by co-receptors of differentiation^14^ and myeloid differentiation protein. TLR2 and TLR4 have been reported that play an important role in behavioral hypersensitivity of neuropathic pain potentially^15–19^. Loss of TLR4 function showed that nerve injury induced behavioral hypersensitivity. TLR4 leads to recruitment of adaptor proteins that determine the downstream signaling pathway in knockout and mutant mice^20–22^. However, the mechanisms regulated by TLR4 in peripheral nerve degeneration and regeneration remain unidentified. Our study will help us to better understand the response of TLR4 to WD and outline the mechanisms by which WD regulates nerve injury and regeneration. Here, we explored the functions of TLR4 on cell migration and proliferation, cell apoptosis, and signal pathways in cultured SCs *in vitro*.

## Materials and Methods

### Animal models of sciatic nerve injury

Male Sprague-Dawley (SD) rats were used. The weight of the rats was 180–200 g. We bought from Animal Center of Nantong University. To undergo sciatic neurectomy, we used total 180 animals, we randomly divided the rats into 5 groups (6 rats/group) (Table 1). All assays were performed in triplicate. The rats were provided by the Experimental Animal Center of Nantong University ((Su) 2014-0001). In this study, all animal injury models were conducted according to the NIH Guidelines for the Care and Use of Laboratory Animals (2011). The animal test protocols were verified by the Key Laboratory of Neuroregeneration, Nantong University Guidelines for the Care and Use of Laboratory Animals. The Institutional Animal Care and Use Committee (IACUC) of Nantong University approved this study (No. 20150820-001). The methods were performed in accordance with the Key Laboratory of Neuroregeneration Guidelines. All rats were anesthetized using complex narcotics (42 mg/kg magnesium sulfate, 85 mg/kg trichloroacetaldehyde monohydrate, and 17 mg/kg sodium pentobarbital). We identified and exposed the rat sciatic nerve on the lateral aspect of the mid-thigh. Then, we cut the sciatic nerve and excised a 1 cm segment. We used one group of rats as the 0 w group immediately and other groups at 1, 2, 3, 4 w following surgery. We used 0 w animals underwent sham operations.

**Table 1:** Number of adult SD rats used

Method	Number				
	0w	1w	2w	3w	4w
Real time PCR	6	6	6	6	6
Western Blot	6	6	6	6	6
Repeated three times					
Sum	180				

### Schwann cells (SCs) primary culture

All rats were bought from the Animal Center of Nantong University (Table 2). Schwann cells were isolated from the sciatic nerves after the rats were sacrificed. The sciatic nerves were dissected from 0–3-day-old SD rats, and minced, incubated in 3 mg/ml collagenase for 30–40 min at 37 °C, followed by trypsinization at 37 °C for 8–10 min. Cells were then cultured in plastic dishes coated with poly-L-lysine, and grown in Dulbecco’s modified Eagle’s medium (DMEM) supplemented with 10% fetal bovine serum (FBS), 100 IU/ml penicillin, 100 g/ml streptomycin (Sigma Aldrich, St Louis, MO, USA) at 37 °C in a humidified atmosphere of 5% CO_2_. Primary cultures were then treated with cytosine arabinoside at 10 μM. The viable fibroblasts were eliminated by complement cleavage of polyclonal anti-Thy1.1 antiserum (Sigma, St Loui, MO) and rabbit complement (Invitrogen, Carisbad, CA). The final cells consisted of 98% SCs, and determined by immunofluorescence for S100 which is a specific SC marker.

**Table 2:** Number of neonate SD rats used.

Method	Number	
	TLR4 knockdown	TLR4 overexpression
Immunohistochemistry	14	
Transfection validation (Real time PCR)	7	7
Transfection validation (Western Blot)	7	7
Annexin V-FITC Apoptosis Detection	7	7
Cell-LightTM EdU DNA Cell Proliferation	7	7
Transwell cell migration	7	7
Analysis of related factors^*^ (Real-time PCR)	7	7
Analysis of related proteins^+^ (Western Blot)	7	7
Repeated three times		
Sum	336	

^*^Includes TLR4, bax (bcl-associated X protein), bcl2 (B-cell lymphoma 2), bFGF (basic fibroblast growth factor), NF2 (Neurofibromin 2), NT3 (Neurotrophin 3), PKCα (protein kinase C, alpha), GAPDH.

^+^Includes TLR4, AKT, p-AKT, beta-catenin, c-fos, p-c-Jun, c-Jun, ERK, p-ERK, PRKCa and beta-actin.

### TLR4 siRNA interference in Schwann cells

We used three different TLR4 siRNAs for small RNA interference (siRNA) experiment (Table 3). The TLR4 siRNAs were synthesized by Integrated Biotech Solutions, Shanghai, China. SCs were cultured and then transfected with TLR4 siRNAs using the transfection reagent of Lipofectamine RNAi MAX (Invitrogen, Carlsbad, CA, USA). NC, the negative control that transfected with the non-specific sequence. We repeated the experiments three times according to the manufacturer’s instructions.

**Table 3:** TLR4-siRNA primers.

Gene	Sequence
NC	F: 5′ UUCUCCGAACGUGUCACGUTT 3′
	R: 5′ ACGUGACACGUUCGGAGAATT 3′
1	F: 5′GGUAAAGAAUUUAGAAGAAGG 3′
	R: 5′UUCUUCUAAAUUCUUUACCAG 3′
2	F: 5′ UGUUAUAUAUACAAGACUAUC 3′
	R: 5′ UAGUCUUGUAUAUAUAACAGG 3′
3	F: 5′ GCAAUUCUUUCAAAGACAACA 3′
	R: 5′ UUGUCUUUGAAAGAAUUGCCA 3′

### TLR4 overexpression in cultured Schwann cells

We cultured SCs in DMEM medium (GIBCO, Grand Island, NY) with 10% fetal calf serum, 100 IU/ml penicillin and 100 g/ml streptomycin at 37 °C under humidified 5% CO_2_. Then the SCs were determined by immunofluorescence with S100, and the final cell culture consisted of 98% SCs. We constructed the TLR4 overexpression plasmid pcDNA3.1-TLR4. Then, we transfected the mixture of the pcDNA3.1-TLR4 plasmid and DNA Transfection Reagent or the empty vector and the DNA Transfection Reagent into SCs for 48 hr. After TLR4 overexpression, we performed real-time qPCR and Western blot analysis.

### Analysis of real-time quantitative PCR

We used Trizol regent to extract total RNA and cDNA Reverse Transcription Kit to synthesize cDNA. After that, we used 7300 Real-Time PCR System to perform real-time quantitative PCR analysis (Table 4). We repeated this analysis three times and ran the PCR reactions three times. We used comparative Ct to analyze the threshold (Ct) value of real-time qPCR data. The differences were considered statistically significant (P < 0.05) of the data were analyzed.

**Table 4:** Primers used in real-time PCR analysis.

Gene	Sequence
TLR4	F: 5′ ACCAGGAAGCTTGAATCCCTG 3′
	R: 5′ CCAGCCACTGAAGTTGTGAG 3′
bax	F: 5′ TGCAGAGGATGATTGCTGAC 3′
	R: 5′ GATCAGCTCGGGCACTTTAG 3′
bcl2	F: 5′ GCAGAGATGTCCAGTCAGC 3′
	R: 5′ CCCACCGAACTCAAAGAAGG 3′
bFGF	F: 5′ CCCGCACCCTATCCCTTCACAGC 3′
	R: 5′ CACAACGACCAGCCTTCCACCCAAA 3′
NF2	F: 5’ CTGGGATTGGGTTCATGGGTGGAT 3′
	R: 5′AGGAAGCCCGAGAAGCAGAGCG 3′
NT3	F: 5′ GACAAGTCCTCAGCCATTGACATTC 3′
	R: 5′ CTGGCTTCTTTACACCTCGTTTCAT 3′
PKCα	F: 5’ GAACACATGATGGACGGGGTCACGAC 3′
	R: 5′ CGCTTGGCAGGGTGTTTGGTCATA 3′
GAPDH	F: 5′ TGGAGTCTACTGGCGTCTT 3′
	R: 5′ TGTCATATTTCTCGTGGTTCA 3′

### Analysis of Western blot

We homogenized SCs and injured nerve samples in protein lysis buffer to extract protein. The lysis buffer contains protease inhibitors. After that, protein expression was analyzed with antibodies against TLR4, AKT, p-AKT, beta-catenin, c-fos, p-c-Jun, c-Jun, ERK, p-ERK, PRKCa and beta-actin. The images were scanned with GS800 Densitometer Scanner. Then, we used PDQuest 7.2.0 software to analyze the optical density. We used beta-actin as a control to normalize the protein levels. We analyzed the Western blot data and considered the differences statistically significant (P < 0.05). In these analyses, we analyzed all samples in three independent experiments.

### Analysis of flow cytometric

The extent of SC apoptosis was measured using Annexin V-FITC Apoptosis Detection Kit (Beyotime Institute of Biotechnology, China) according to the manufacturer’s instruction. SCs were collected for flow cytometric analysis after washed with PBS. We added 5 μl FITC-labeled annexin V and 195 μl binding buffer. Then incubated the cells for another 10 min at room temperature. After that, we added 10 μl propidium iodide and continued incubation for 10 min on ice in the dark. Then, we used FACScan flow cytometry to measure the apoptotic cells.

### Assay of cell proliferation

We cultured and plated SCs onto poly-L-lysine-coated plates with a density of 2 × 10^5^ cells/ml. SCs were transfected and then cell proliferation was assayed. We added EdU to the cells and the cells were incubated for another 2 hours. SCs were fixed with formaldehyde for 0.5 hr. After SCs were labeled, the cell proliferation was assayed by Cell-Light^TM^ EdU DNA Cell Proliferation Kit. A DMR fluorescence microscope (Leica Microsystems, Bensheim, Germany) was used to obtain the images of randomly selected fields from which we assayed cell proliferation by the ratio of EdU-positive cells. We performed cell proliferation assays with triplicate wells.

### Assay of cell migration

We used transwell chambers to examine SC migration. Transwell chambers (6.5 mm) with 8-μm pores were used. Here, 100 µL DMEM containing 10^6^ resuspended SCs/ml was transferred to the top transwells chambers and allowed to migrate into the lower chamber. Before the addition of 600 µL complete medium into the chambers, the SCs were allowed to migrate in 5% CO_2_. Cells of each membranes adhered to the bottom of the transwell chambers surface. We stained these SCs with 0.1% crystal violet, then used DMR inverted microscope (Leica Microsystems, Bensheim, Germany) to image and count the cells. The cell migration assays used triplicate wells.

### Immunohistochemistry

Immunohistochemistry was used to visualize the location of TLR4 and S100 in cultured SCs. We immunostained these SCs with anti-S100, a specific SC marker. We used 4% paraformaldehyde to fixed SCs and 30% sucrose solution to dehydrate SCs. Then, we obtained 12-µm thick cryostat sections. We rinsed the slides in PBS, permeabilized the slides in 5% goat serum, 0.3% Triton-X and 1% BSA in PBS, and then the slides were stained. Rabbit anti-S100 (1:400, Abcam) and anti-TLR4 (1:50, Novusbio) antibody were incubated with the slides at 4 °C for 12 hr. We incubated the sections with goat anti-rabbit IgG Cy3 and goat anti-mouse IgG Alexa Fluor 488 at room temperature for 2 hr. After that, the slides were stained with Hoechst 33342. We used fluorescence microscope observed all samples.

### Statistical analysis

We used SPSS 15.0 for windows to perform the statistical analyses, and Student’s t-test for analysis of the two-group comparisons. All data were expressed in this study as the mean ± SD. We considered P < 0.05 as statistically significant.

## Results

### TLR4 is expressed in injured sciatic nerves and cultured Schwann cells (SCs)

**Figure 1:**
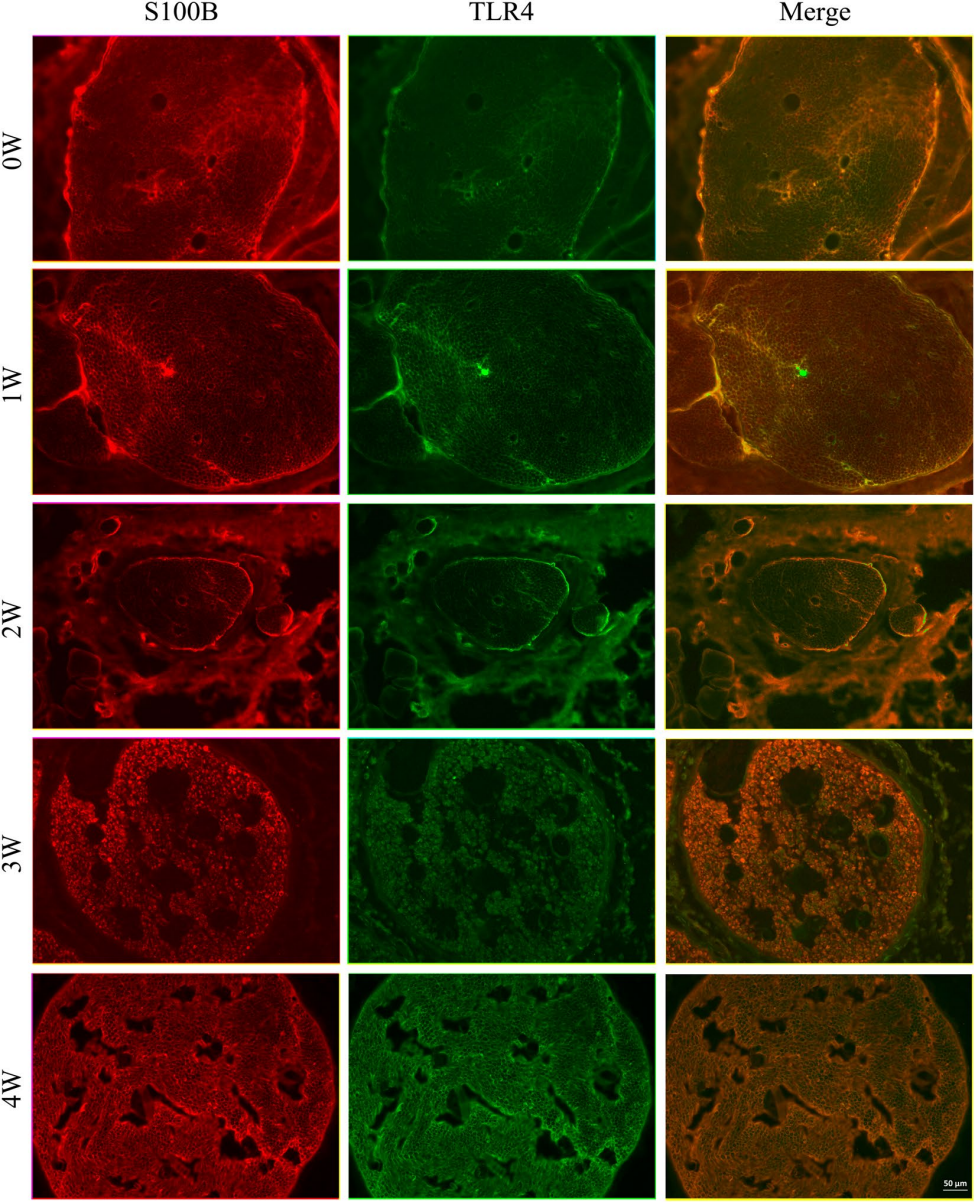
Immunohistochemistry analysis of TLR4 expressions after rat sciatic nerve injury (0 W, 1 W, 2 W, 3 W and 4 W). Immunofluorescence staining for distal sciatic nerve stumps of rats at indicated time intervals Immunostained for S100, TLR4 and their overlay. S100 was used as SC specific marker. (Bar = 50 μm).

**Figure 2:**
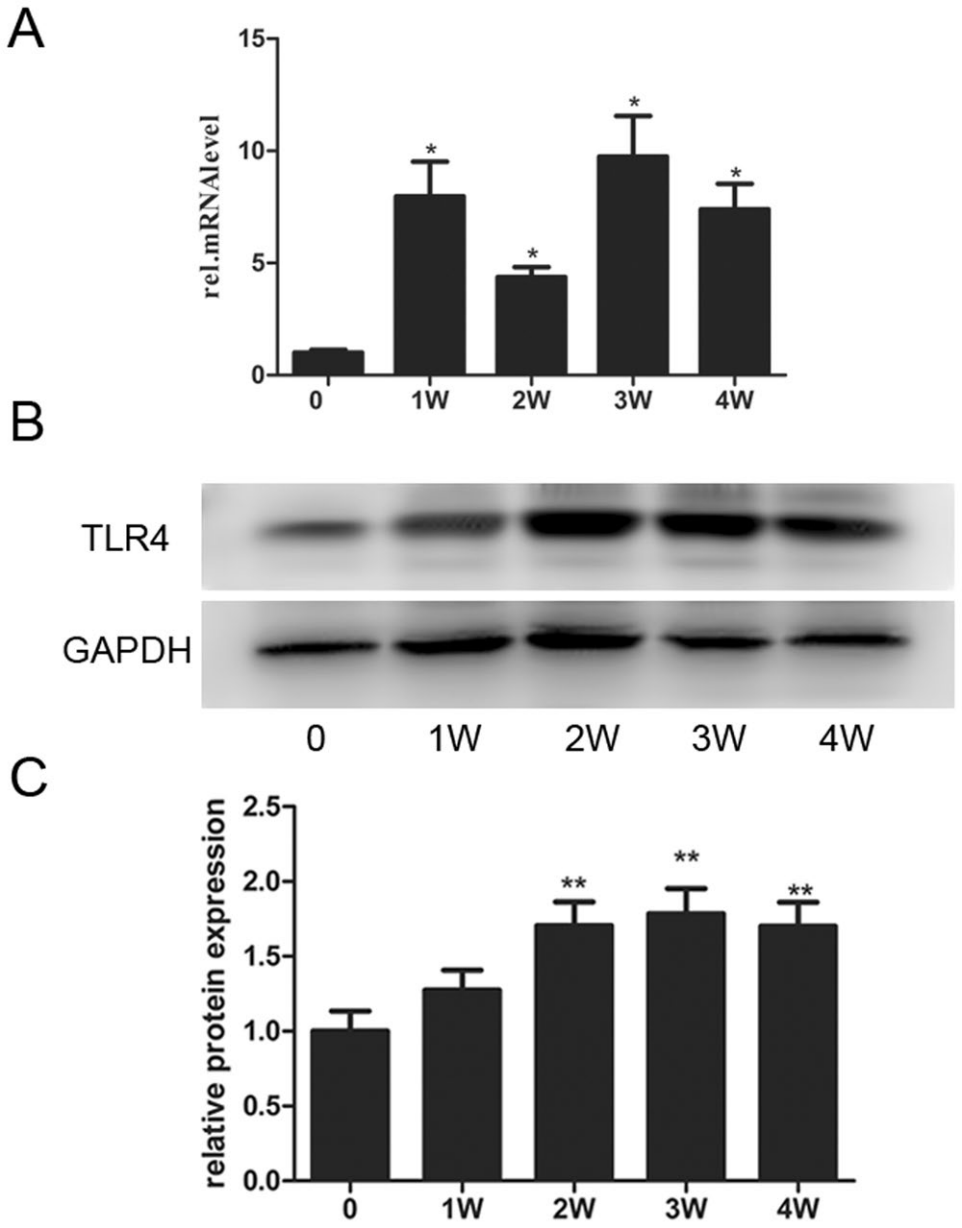
TLR4 expression in the injured distal sciatic nerve stumps of rats. (**A**) Real-time qPCR analysis of TLR4 expression in the sciatic nerve after injury at 0, 1, 2, 3 and 4 w after surgery using beta-actin as the normalizer. The average of three independent experiments is shown ± SEM (*p < 0.05). (**B**) Western blot analysis of the TLR4 expression in the injured sciatic nerve at 0, 1, 2, 3 and 4 w after surgery. Beta-actin levels were used as a control. (**C**) Relative expression of TLR4 was analyzed by Western blot analysis. In order to show the order of sample, we left an empty lane between 0 d protein and the others.

**Figure 3:**
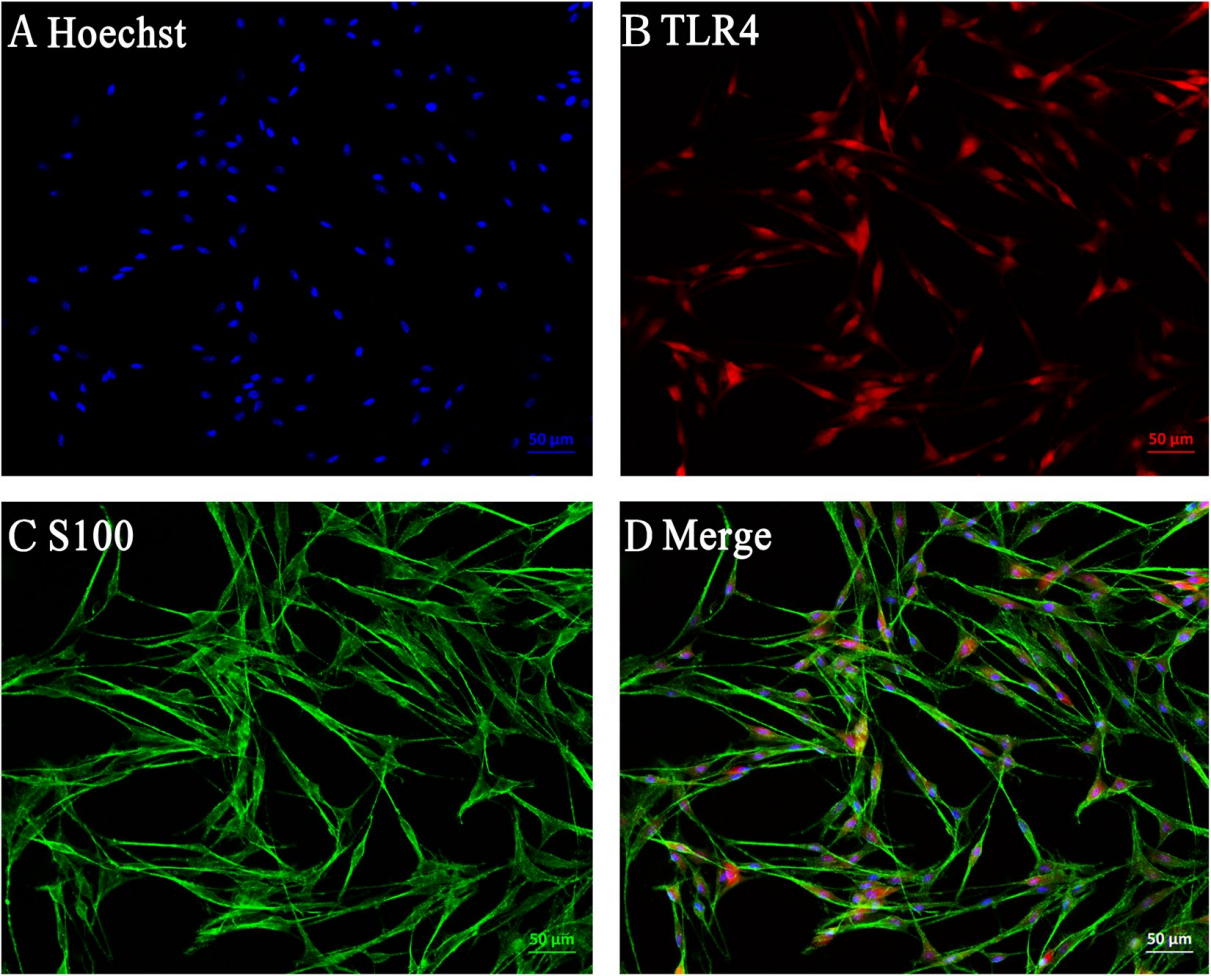
TLR4 is located in Schwann cells. (**A**) Immunofluorescence staining of cultured Schwann cells (SC) for Hoechst (blue). (**B**) Immunofluorescence staining of cultured SCs for S100 (green). (**C**) Immunofluorescence staining of cultured SCs for TLR4 (red). (**D**) Immunofluorescence staining of cultured SCs for Hoechst (blue), S100 (green) and TLR4 (red) overlay. S100 was used as a SC-specific marker. (200×, Bar = 50 μm). The experiment was repeated three times.

The expression levels of TLR4 at specified time points after sciatic nerve injury were first verified by Immunohistochemisty, Western blot and real-time qPCR. Immunohistochemisty was used to visualize the localization of TLR4 and S100B within the sciatic nerve after injury. The SCs were immunostained using anti-S100B. TLR4 and S100B were colocalized in SCs, indicating that TLR4 is expressed in the rat sciatic nerve (Fig. 1). Our previous research reported on the expression changes and bioinformatic analysis of Wallerian degeneration after sciatic nerve injury in rat. We used signal flow to analyze the microarray results and found out the expression changes after sciatic nerve injury in rat. Based on the bioinformatics analysis and key time points of WD, these assays were applied to investigate the TLR4 expression level at 0, 1, 2, 3 and 4 w after injury. The results revealed that the TLR4 expression level was increased from 1 to 4 weeks after injury. Beta-actin levels were used as a control (Fig. 2). To further verify the expression of TLR4 in SCs, we first cultured and purified Schwann cells (≥95%), and then we used immunohistochemistry to analyze the location of S100 and TLR4 in cultured SCs. Here, we immunostained SCs with anti-S100. The immunostained cells showed that TLR4 and S100 co-localized in Schwann cells (Fig. 3) (Supplementary Data Fig. S2). Our data indicate that TLR4 is expressed in rat sciatic nerves after injured and cultured SCs. Experiments were assayed in triplicate (*P < 0.05, vs. day 0). All data in this study were expressed as the mean ± SD and were analyzed by one-way analysis and Scheffe’s post test.

### TLR4 affects SC proliferation

**Figure 4:**
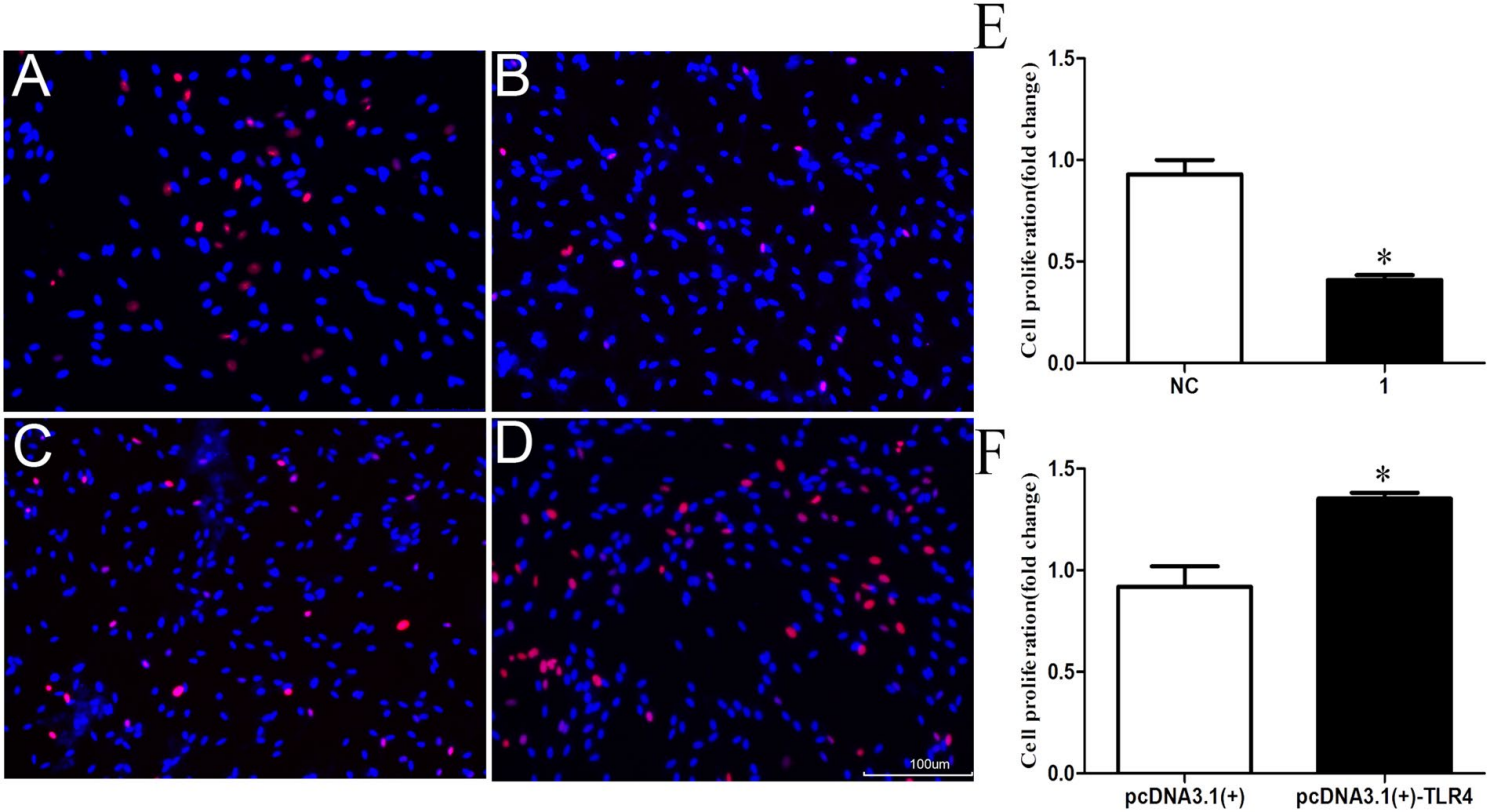
TLR4 knockdown and overexpression affect SC proliferation. (**A**,**B**) Silencing of TLR4 by transfection with TLR4 siRNA inhibited SC proliferation compared with the negative control that transfected with the non-specific sequence (NC). (**C**,**D**) Overexpression of TLR4 by transfection with the pcDNA3.1-TLR4 plasmid induced SC proliferation compared with that of the the negative control. (**E**) Relative number of cells in TLR4-knockdown SCs. (**F**) Relative number of cells in TLR4-overexpressed SCs. Blue represents the nucleus of SCs which we cultured, red represents the number of cells that proliferated in EdU, and the histogram is the data graph of EdU. The average of three independent experiments is shown ± SEM (*P < 0.05). Bar = 50 μm.

To analyze the function of TLR4 in nerve injury and regeneration, the TLR4 siRNAs, the pcDNA3.1-TLR4 plasmid and negative controls were transfected to cultured SCs (Supplementary Data Fig. S1). The assay results indicated that the proliferation of SCs transfected with TLR4 siRNA was smaller than that of the control, whereas the proliferation rate of pcDNA3.1-TLR4-transfected cells was greater than that of the control (Fig. 4). In this data, blue represents the nucleus of SCs which we cultured, red represents the number of cells that proliferated in EdU, and the histogram is the data graph of EdU. Ethynyl deoxyuridine (EdU) is a novel thymidine analogue for labelling dividing cells, detected with a fluorescent azide which forms a covalent bond via the chemistry reaction. These data suggest that the expressions of TLR4 affected the SC proliferation. The data were assayed in triplicate (*P < 0.05).

### TLR4 affects SC migration

**Figure 5:**
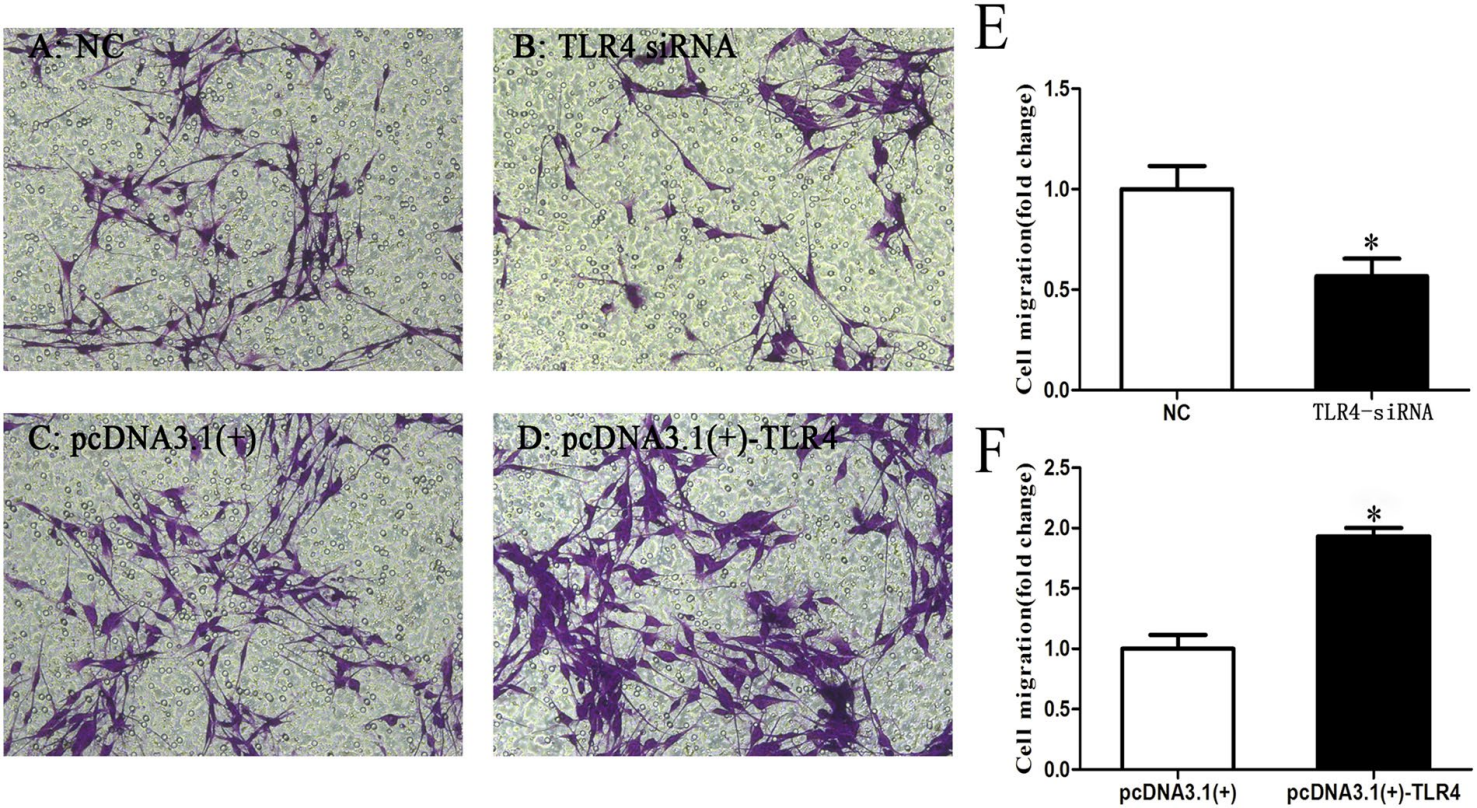
TLR4 knockdown and overexpression affect SC migration. (**A**,**B**) Silencing of TLR4 by transfection with TLR4 siRNA induced SC migration compared with the negative control that transfected with the non-specific sequence (NC). (**C**,**D**) Overexpression of TLR4 by transfection with the pcDNA3.1-TLR4 plasmid inhibited SC migration compared with that of the negative control. (**E**) Relative number of cells in TLR4-knockdown SCs. (**F**) Relative number of cells in TLR4-overexpressed SCs. The average of three independent experiments is shown ± SEM (*P < 0.05).

To further explore the effect of TLR4 on SCs, we analyzed the effect of TLR4 on SC migration. After transfection with TLR4 siRNA, the pcDNA3.1-TLR4 plasmid or a negative control, the data showed that the cell migration rate of the SCs transfected with TLR4 siRNAs was decreased than that of the control cells transfected with the negative control that transfected with the non-specific sequence (NC). By contrast, the cell migration which transfected with pcDNA3.1-TLR4 was greater than that of the control (Fig. 5). Purple represents the migrating cells, and the histogram is the Transwell data graph. Transwell chamber has a permeable membrane with micropores. We put the cells in the upper chamber. Since the polycarbonate membrane is permeable, the composition of the lower culture medium can affect the cells. SCs were attracted to the lower layer through the nutrient factors of the culture medium. We assayed these data in triplicate (*P < 0.05).

### TLR4 affects SC apoptosis

**Figure 6:**
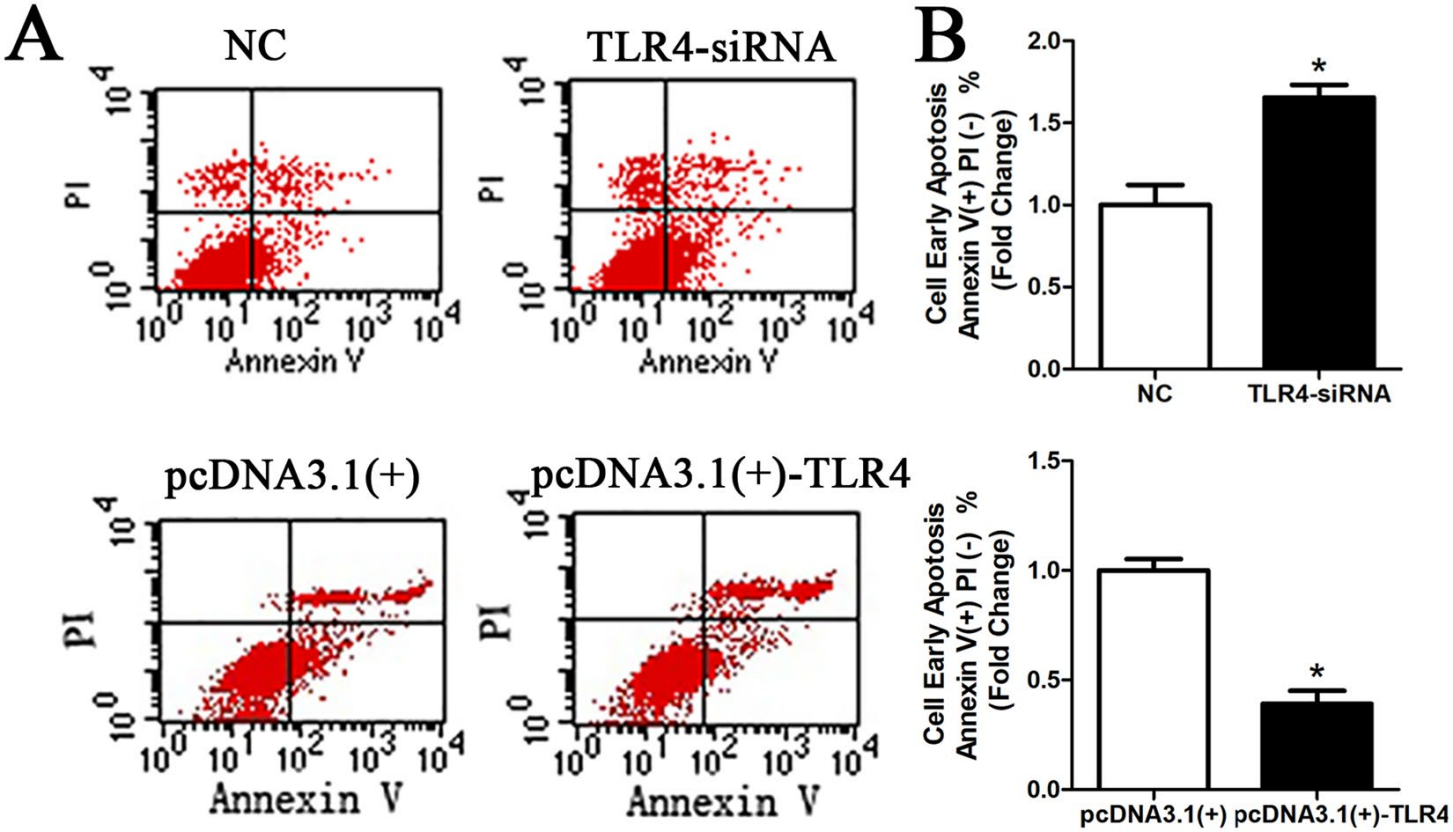
TLR4 knockdown and overexpression affect SC apoptosis. (**A**) Silencing of TLR4 by transfection with TLR4 siRNA induced SC apoptosis compared with the negative control that transfected with the non-specific sequence (NC) and overexpression of TLR4 by transfection with the pcDNA3.1-TLR4 plasmid inhibited SC apoptosis compared with that of the negative control. (**B**) Relative number of cells in TLR4-knockdown SCs and relative number of cells in TLR4-overexpressed SCs. The average of three independent experiments is shown ± SEM (*P < 0.05).

After the cell migration and proliferation assays, we determined the function of TLR4 in SC apoptosis. We cultured SCs and transfected SCs with TLR4 siRNAs, pcDNA3.1-TLR4 plasmid and negative control. We examined the effect of TLR4 siRNA and pcDNA3.1-TLR4 on cell apoptosis. The results showed that the cell apoptosis rate was higher in TLR4 siRNA-transfected cells and lower in pcDNA3.1-TLR4-transfected cells than in the control cells (Fig. 6). Here is the flow cytometry analysis after Annexin V-FITC and propidium iodide (PI) staining. Normal cells are not stained with Annexin V-FITC nor propidium iodide; early apoptotic cells are only stained with Annexin V-FITC; Necrotic cells and advanced apoptotic cells can be simultaneously stained with Annexin V-FITC and propidium iodide (upper right part of the figure). The results indicate that the TLR4 down-regulation induces SCs apoptosis and the TLR4 up-regulation reduces SCs apoptosis (*P < 0.05).

### Altered expression of TLR4 results to a change in gene expression of SCs

**Figure 7:**
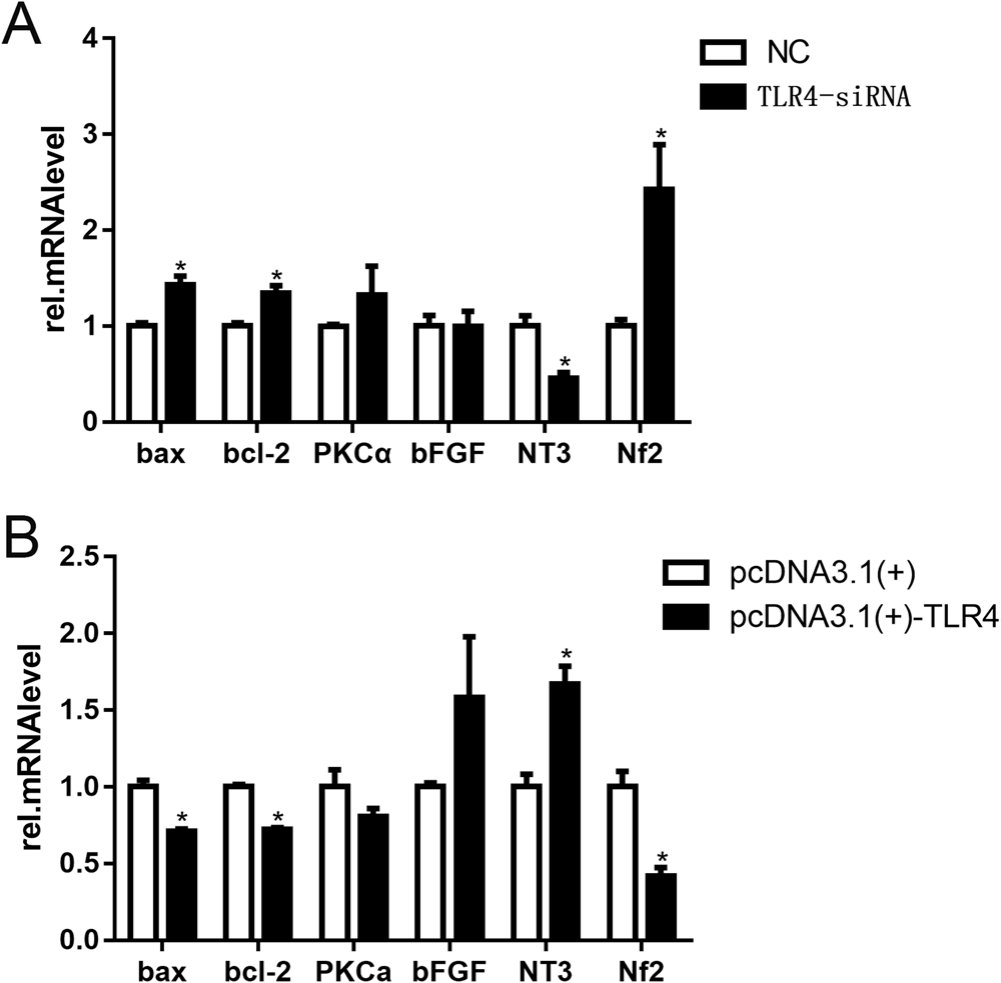
Relative gene expressions in TLR4-knockdown and -overexpressed SCs. (**A**) Real-time qPCR analysis of bax, bcl2, PKCa, bFGF, NT3 and nf2 expression levels after transfection of SCs with TLR4 siRNA for 2 d using beta-actin as the normalizer compared to the levels in the negative control cells (*p < 0.05). The average of three independent experiments is shown ± SEM (*p < 0.05). (**B**) Real-time qPCR analysis of bax, bcl2, PKCa, bFGF, NT3 and nf2 expression levels after transfection of SCs with the pcDNA3.1-TLR4 plasmid for 2 d using beta-actin as the normalizer compared to the levels in the negative control cells (*p < 0.05). The average of three independent experiments is shown ± SEM (*p < 0.05).

To further investigate the functions of TLR4 on gene expression in SCs, we analyzed the expression of bax, bcl2, PKCa, bFGF, nf2 and NT3 after TLR4 overexpression and knockdown in transfected SCs. The real-time PCR data showed that the expression levels of bax, bcl2, PKCa and nf2 were up-regulated in TLR4 knock-down SCs and down-regulated in TLR4 overexpressed SCs. However, the expression levels of bFGF and NT3 were down-regulated in TLR4-knockdown SCs and up-regulated in TLR4-overexpressed SCs (Fig. 7). The data suggest that TLR4 differential expression results in a change in gene expression in SCs (*P < 0.05).

### TLR4 affects the c-Jun, ERK and catenin pathways

**Figure 8:**
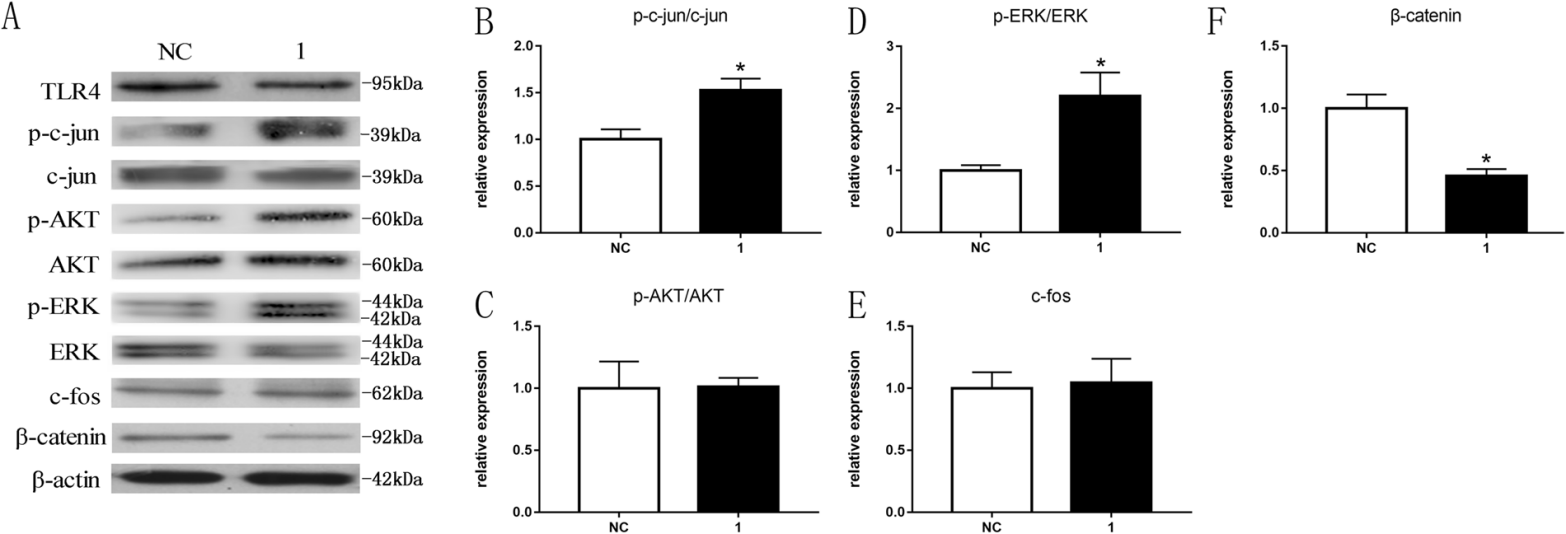
TLR4 affects c-Jun, ERK and catenin but not AKT and c-Fos pathways in TLR4-knockdown SCs. (**A**) Western blot analysis of p-c-Jun/c-Jun, p-AKT/AKT, p-ERK/ERK, c-Fos and beta-catenin expression after transfection of SCs with TLR4 siRNA for 2 d using beta-actin as the normalizer compared to that in the negative control cells. The average of three independent experiments is shown ± SEM. (**B**–**F**) Relative expression of p-c-Jun/c-Jun, p-AKT/AKT, p-ERK/ERK, c-Fos and beta-catenin as shown by Western blot analysis. The average of three independent experiments is shown ± SEM.

**Figure 9:**
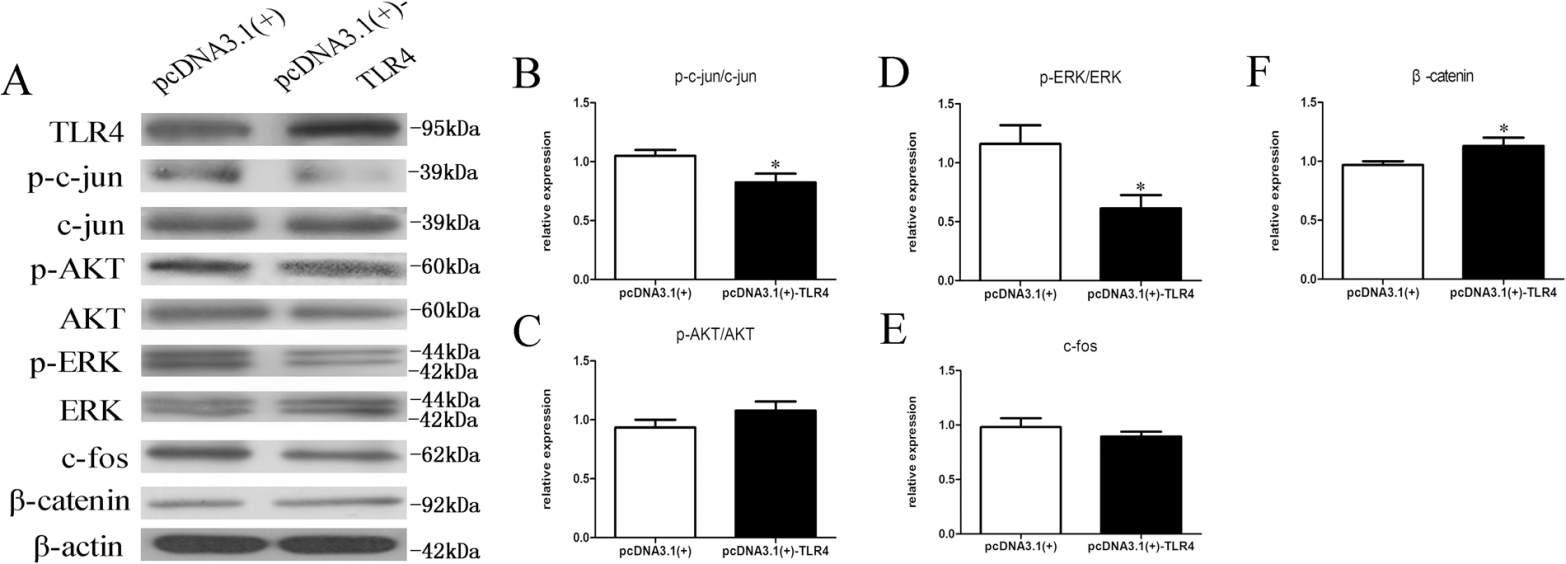
TLR4 affects c-Jun, ERK and catenin but not AKT and c-Fos pathways in TLR4-overexpressed SCs. (**A**) Western blot analysis of p-c-Jun/c-Jun, p-AKT/AKT, p-ERK/ERK, c-Fos and beta-catenin expression after transfection of SCs with the pcDNA3.1-TLR4 plasmid for 2 d using beta-actin as the normalizer compared to that in the negative control cells. The average of three independent experiments is shown ± SEM (*p < 0.05). (**B**–**F**) Relative expression of p-c-Jun/c-Jun, p-AKT/AKT, p-ERK/ERK, c-Fos and beta-catenin as shown by Western blot analysis. The average of three independent experiments is shown ± SEM (*p < 0.05).

As the data showed above, altered TLR4 expression led to changes in the expression of some factors, but how TLR4 expression affected signal pathways in cultured SCs was still unknown. Here, we assayed the expression levels of p-c-Jun/c-Jun, p-ERK/ERK, p-AKT/AKT, c-Fos and beta-catenin and compared them to negative controls. The results showed that the expression of PKCa, c-fos and p-ERK/ERK was significantly affected in TLR4 knock-down SCs (Fig. 8) and TLR4 overexpressed SCs (Fig. 9), while the expression of AKT and c-Fos was not significantly altered (*P < 0.05). All these data suggest that TLR4 may play important roles in regulation of the c-Jun, ERK and catenin but not the AKT or c-Fos pathways in cultured SCs.

## Discussion

Peripheral nerve injury and repair is a process of degeneration and regeneration activated by injury-dependent reactions. During this process, differentially expressed genes are significantly regulated, which is essential for distal stump reinnervation or repair^23–28^. The factors that stimulate the signal pathways and initiate the degeneration and regeneration responses after nerve injury are not clear^29–35^. Therefore, understanding the factors that regulate WD may provide further insights into nerve degeneration and/or regeneration.

Cell death-associated genes, such as the B-cell lymphoma (Bcl)-like protein Fas receptor/ligand receptor (Fasl) and Bcl-associated X protein (Bax) are re-expressed upon nerve injury. Apoptosis is also a mediator of secondary damage after nerve injury^36–39^. In this study, we have identified pro-apoptotic changes in SCs caused by TLR4 knockdown and TLR4 overexpression, including the regulation of anti-apoptotic Bcl-2 and pro-apoptotic Bax as seen through flow cytometric and real-time PCR analyses. Our data have demonstrated that the secondary damage to SCs is associated with changes in TLR4 expression. These changes are important for early factors expression responses to activation and proliferation after injury. SCs release endogenous ligands to recognize and combine with TLR4. TLR4 activation and signal transduction facilitate the activation of the release of multiple factors. There is a possibility that TLR4 can recognize endogenous molecules that are abnormally presented as ‘danger signals’^40–43^.

In this research, we reported the effect of TLR4 in cultured Schwann cells *in vitro*. TLRs are the key mediators that respond to injury-induced endogenous ligands. Some TLRs alert the host to tissue damage after having been activated by the molecules released from the injuried tissue. SCs have been reported to express several TLR family members, including TLRs^18,19^. TLRs appear to be recruited by TLR4 in early peripheral nerve injury. TLRs were produced by injured axons, necrotic cells or extracellular matrix which have been reported in regulating WD and nerve degeneration/regeneration after nerve injury^18–20^. TLR4 may faster clearance of the degenerating myelin and recovered earlier axonal degeneration, locomotor function of rats sciatic nerves^20–23^. Here, we investigated the effect of TLR4 in cultured SCs. The data showed that TLR4 expression affected SC proliferation, migration and apoptosis. Our data provide strong evidence that TLR4 plays an important role in SC behavior changes *in vitro*^18–23^.

The mechanism by which the TLR4 gene is regulated in nerve injury during WD is not yet understood. Therefore, whether TLR4 is responsible for nerve repair and regeneration in cultured SCs remains to be investigated. SCs may have an effect on axon growth through their released factors^44–46^. However, whether these factors act in response to TLR4 activation or not, it remains to be determined.In our model, we have explored our data in the presence of SCs. Previous studies have reported that TLR4 play roles in the maintenance and initiation the hypersensitivity phases in neuropathic models. TLR4 is expressed in astrocytes, microglia and neurons. A loss of TLR4 function results in attenuated behavioral hypersensitivity induced by nerve injury. Accelerating Wallerian degeneration after injury might shorten the period of peripheral nerve regeneration. Studies have shown that immune cells are activated by endogenous ligands after tissue damage^47,48^. Here we reported the effect of TLR4 on cultured Schwann cells *in vitro*. Studies needed to further clarify the role of TLR4 *in vivo* after peripheral nerve injury and will be pursued in our future research.

Taken together, our data suggest that TLR4 may be activated in peripheral nerve regeneration. The study helps to elucidate the importance of TLR4 for nerve regeneration and may provide the basis for future therapeutic strategies to treat nerve injury patients.

### Ethical approval and consent to participate

All animal tests were conducted in accordance with the US National Institutes of Health’s Guide for the Care and Use of Laboratory Animals and by the Key Laboratory of Neuroregeneration Guidelines for the Care and Use of Laboratory Animals. The Institutional Animal Care and Use Committee of Nantong University approved all protocols used in this study.

## Electronic supplementary material

Supplementary Data S1

Supplementary Data S2
