# Supplementary material for: Toll-Like Receptor 4 (TLR4) Expression Affects Schwann Cell Behavior in vitro
Source: Sci Rep. 2018 Jul 25;8:11179. doi: 10.1038/s41598-018-28516-5 (PMC6060163; doi:10.1038/s41598-018-28516-5)
Supplement: Supplementary file 1 — Supplementary Data S1 [file 41598_2018_28516_MOESM1_ESM.pdf]

Toll-Like Receptor 4 (TLR4) Expression Affects Schwann Cell Behavior *in vitro*

Huanhuan Zhang[#], Zhiwei Shao[#], Yun Zhu, Lingyu Shi, Zhihao Li, Rui Hou, Chunwang Zhang, Dengbing Yao[*]

School of Life Sciences, Key Laboratory of Neuroregeneration, Co-innovation Center of Neuroregeneration, Nantong University, Nantong, Jiangsu 226019, P.R. China.

[#]These authors contributed equally to this work.

[*]Corresponding author: Dengbing Yao, School of Life Sciences, Key Laboratory of Neuroregeneration, Co-innovation Center of Neuroregeneration, Nantong University, No. 9 Seyuan Road, Nantong, Jiangsu 226019, P.R. China. Tel.: +86-513-85012826; Fax: +86-513-85012810. E-mail: yaodb@ntu.edu.cn

Supplementary Data Fig. S1

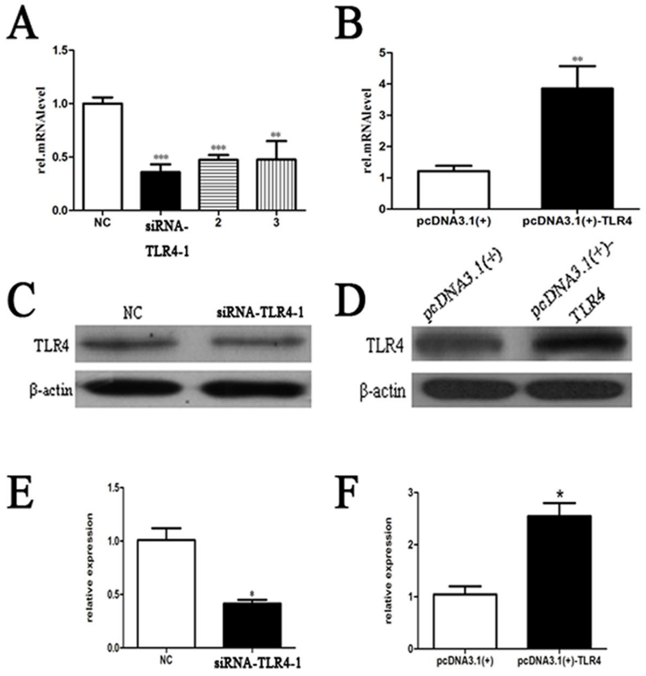

Supplementary Data Fig. S1. TLR4 expressions in cultured SCs *in vitro*. (A), (B). Real-time PCR analysis of TLR4 siRNA 1, 2, 3 and pcDNA3.1-TLR4 plasmid were transfected to cultured SCs. (C), (D). Western Blot analysis of TLR4 siRNA 1 and pcDNA3.1-TLR4 plasmid transfected to cultured SCs. (E), (F). Relative expressions for Western blot analysis of TLR4 siRNA 1 and pcDNA3.1-TLR4 plasmid transfected to cultured SCs. The average of three independent experiments is shown ±SEM (*, $p<0.05$).
