# Supplementary material for: Toll-Like Receptor 4 (TLR4) Expression Affects Schwann Cell Behavior in vitro
Source: Sci Rep. 2018 Jul 25;8:11179. doi: 10.1038/s41598-018-28516-5 (PMC6060163; doi:10.1038/s41598-018-28516-5)
Supplement: Supplementary file 2 — Supplementary Data S2 [file 41598_2018_28516_MOESM2_ESM.pdf]

Toll-Like Receptor 4 (TLR4) Expression Affects Schwann Cell Behavior *in vitro*

Huanhuan Zhang#, Zhiwei Shao#, Yun Zhu, Lingyu Shi, Zhihao Li, Rui Hou, Chunwang Zhang, Dengbing Yao*

School of Life Sciences, Key Laboratory of Neuroregeneration, Co-innovation Center of Neuroregeneration, Nantong University, Nantong, Jiangsu 226019, P.R. China.

#These authors contributed equally to this work.

*Corresponding author: Dengbing Yao, School of Life Sciences, Key Laboratory of Neuroregeneration, Co-innovation Center of Neuroregeneration, Nantong University, No. 9 Seyuan Road, Nantong, Jiangsu 226019, P.R. China. Tel.: +86-513-85012826; Fax: +86-513-85012810. E-mail: yaodb@ntu.edu.cn

Supplementary Data Fig. S2

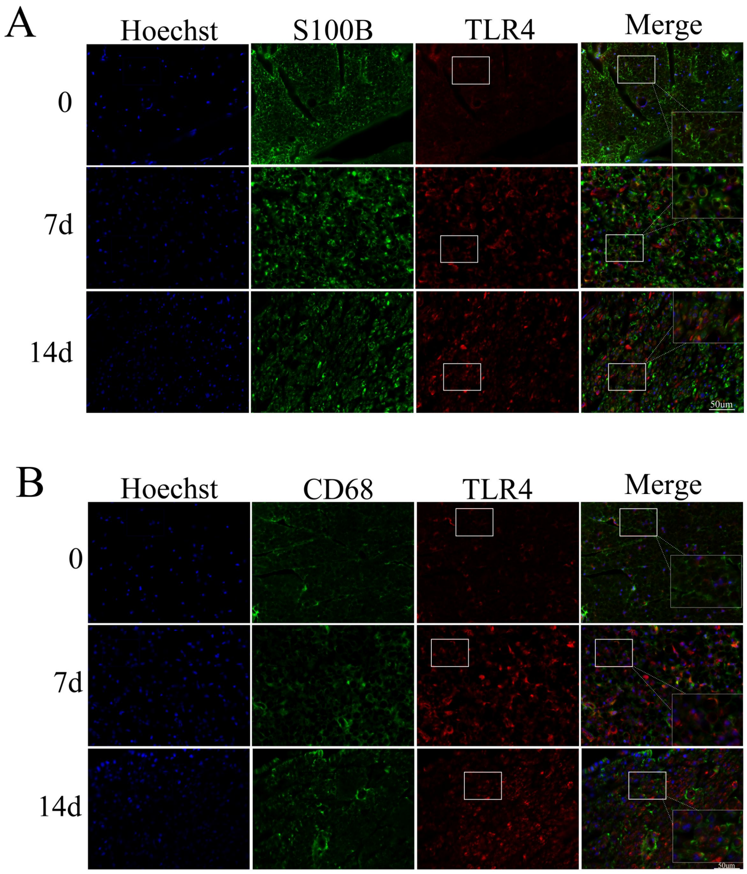

Supplementary Data Fig. S2. TLR4 is expressed in injured sciatic nerves. (A) Immunofluorescence staining of injured sciatic nerves for Hoechst (blue), S100 (green), TLR4 (red) and overlay at 0, 7, 14d. (B) Immunofluorescence staining of injured sciatic nerves for Hoechst (blue), CD68 (green), TLR4 (red) and overlay at 0, 7, 14d. S100 was used as a SC-specific marker. (200×, Bar = 50 μm). The experiment was repeated three times.
